# 698 Pediatric Hand Burn Contractures: A Systematic Review and Meta-analysis

**DOI:** 10.1093/jbcr/iraf019.327

**Published:** 2025-04-01

**Authors:** Tiffany Jeong, Ana Reis, Hilary Liu, José Arellano, Justine Kim, Teun Teunis, Francesco Egro

**Affiliations:** University of Pittsburgh School of Medicine; University of Pittsburgh Medical Center; University of Pittsburgh Medical Center; University of Pittsburgh Medical Center; University of Pittsburgh Medical Center; University of Pittsburgh Medical Center; University of Pittsburgh Medical Center

## Abstract

**Introduction:**

Contractures continue to pose a significant challenge in both post-acute care and post-secondary reconstruction of hand burns. For pediatric patients, growth presents a new dimension of strain to skin grafts that may exacerbate contracture formation. However, the burden of post-burn hand contractures in pediatric patients remains unclear in the literature. This study aims to elucidate post-hand burn contracture occurrence and recurrence in the pediatric population.

**Methods:**

A systematic review and meta-analysis was conducted and reported according to PRISMA guidelines, and the protocol registered on PROSPERO. The results were limited to articles that reported on contracture as a complication with extractable data on pediatric patients. Meta-analysis was conducted using a random-effects model.

**Results:**

Of the 2494 retrieved articles, 46 were confirmed to be relevant to the subject matter and proceeded to full article review. Of these, 16 contained sufficient data for analysis. Six articles, comprising 292 hands, reported on acute treatment and initial contracture occurrence. The remaining 10 articles, with 425 hands, discussed secondary reconstruction for contracture release.

Acute coverage was established with split thickness skin grafts (STSG, n=171), full-thickness skin grafts (FTSG, n=119), or a combination of both (n=3). The contracture rate for acute pediatric hand burns was 0.15 [95% CI: 0.06, 0.23].

Reconstructive contracture release patients received the following surgical interventions: FTSG (n=241), local flaps (n=110), z-plasty (n=57), STSG (n=39), external fixators (n=6), and phalangeal amputation (n=5). The contracture recurrence rate for pediatric hand burns was 0.12 [95% CI: 0.01, 0.25]

**Conclusions:**

The results of our meta-analysis suggest key insights into the occurrence and recurrence of hand burn contractures in children. The initial contracture rate after acute skin graft treatment is relatively low, suggesting some effectiveness of current methods. However, the recurrence after secondary reconstruction highlights ongoing management challenges. Improved techniques and long-term care strategies are needed, and further research is essential to optimize treatment protocols and reduce contractures in pediatric patients.

**Applicability of Research to Practice:**

Burn reconstruction of key functional areas often occurs in multiple stages and can require long term follow up with patients for successful management. With information on the occurrence and recurrence of pediatric hand contractures, we can better inform our patients of the expected outcomes after surgery.

**Funding for the Study:**

N/A

